# Medical school clinical placements – the optimal method for assessing the clinical educational environment from a graduate entry perspective

**DOI:** 10.1186/s12909-017-1113-y

**Published:** 2018-01-05

**Authors:** Sarah Hyde, Ailish Hannigan, Tim Dornan, Deirdre McGrath

**Affiliations:** 1 Graduate Entry Medical School, University of Limerick, Limerick, Ireland; 2 School of Medicine, Dentistry and Biomedical Sciences, Queens University, Belfast, UK

**Keywords:** Clinical education, DREEM, Manchester clinical placement index

## Abstract

**Background:**

Educational environment is a strong determinant of student satisfaction and achievement. The learning environments of medical students on clinical placements are busy workplaces, composed of many variables. There is no universally accepted method of evaluating the clinical learning environment, nor is there consensus on what concepts or aspects should be measured. The aims of this study were to compare the Dundee ready educational environment measure (DREEM - the current de facto standard) and the more recently developed Manchester clinical placement index (MCPI) for the assessment of the clinical learning environment in a graduate entry medical student cohort by correlating the scores of each and analysing free text comments. This study also explored student perceptionof how the clinical educational environment is assessed.

**Methods:**

An online, anonymous survey comprising of both the DREEM and MCPI instruments was delivered to students on clinical placement in a graduate entry medical school. Additional questions explored students’ perceptions of instruments for giving feedback. Numeric variables (DREEM score, MCPI score, ratings) were tested for normality and summarised. Pearson’s correlation coefficient was used to measure the strength of the association between total DREEM score and total MCPI scores. Thematic analysis was used to analyse the free text comments.

**Results:**

The overall response rate to the questionnaire was 67% (*n* = 180), with a completed response rate for the MCPI of 60% (*n* = 161) and for the DREEM of 58% (*n* = 154). There was a strong, positive correlation between total DREEM and MCPI scores (*r* = 0.71, *p* < 0.001). On a scale of 0 to 7, the mean rating for how worthwhile students found completing the DREEM was 3.27 (SD 1.41) and for the MCPI was 3.49 (SD 1.57). ‘Finding balance’ and ‘learning at work’ were among the themes to emerge from analysis of free text comments.

**Conclusions:**

The present study confirms that DREEM and MCPI total scores are strongly correlated. Graduate entry students tended to favour this method of evaluation over the DREEM with the MCPI prompting rich description of the clinical learning environment. Further study is warranted to determine if this finding is transferable to all clinical medical student cohorts.

## Background

An educational environment, as it is perceived by students, is a strong determinant of student satisfaction and achievement [1] and has an effect on many aspects of student behaviour, for example professional [2], and moral [3] development. The learning environment of medical students on clinical placement is a busy, sometimes chaotic workplace and is composed of many variables including the student, curriculum, teachers, patients, allied health professionals, outpatient clinics, operating theatres, as well as social, emotional and psychological factors, all of which may impact on student learning. As it has been shown that detecting differences between student’s actual and ideal learning environments can lead to changes in a schools programme and thus an improved student experience [4], measuring the quality of educational environments is therefore important [5].

The optimal method of measuring quality in a clinical educational environment remains unknown [5]. Because educational environments are composed of multiple variables, there has been disagreement over what aspects should be measured [6]. There is therefore no universally accepted method of evaluating the clinical learning environment, nor is there even consensus on what concepts or aspects should be measured [7]. The relatively large number of instruments available to measure the educational environment suggests that there may be uncertainty over the item validity and sampling validity of these instruments [8]. The current de-facto standard used by many institutions is the Dundee Ready Education Environment Measure (DREEM). The DREEM was designed by a Delphi panel of medical educators [9]. It has been widely translated and there is much published literature on its use [10–12]. However, questions have been raised over its psychometric properties [11], and its internal consistency [13]. Another important issue is that, in the authors’ opinion, it appears more suited to assessing the learning environment in a preclinical setting, rather than that of a complex working clinical site. The most recent review of instruments available to measure medical educational environments reports there being limited validity evidence for any of the measurement tools currently in use [14]. Furthermore, a review looking at the underlying theoretical framework informing the design of assessment instruments identified eleven instruments including the DREEM, and found that none were grounded in educational theory. It has been argued that an instrument grounded in educational theory would be preferable [7].

The Manchester Clinical Placement Index (MCPI) [15] is a newer, designed for purpose instrument which has attempted to rectify the lack of theoretical grounding in previous instruments, and to address the unique aspects of a clinical working environment, potentially making it a more valid instrument for use in this setting. The MCPI is based on Experience-Based-Learning in Communities-of-Practice educational theory, and has a smaller number of items, 8 compared with 50 items in the DREEM. The psychometric properties and validity of the MCPI has previously been explored [16]. A study by Kelly, Bennett et al. has shown equivalent discrimination between placements as the DREEM when the MCPI was used to measure the clinical learning environment of direct entry students in a medical degree programme [16, 15] That study however did not explore acceptability to students or the content of the free text comments.

A graduate entry programme is a suitable environment for assessing the face validity (the extent to which an instrument seems to measure what it sets out to measure) of these instruments. To date the clinical educational environment as experienced by graduate entry medical students has not been assessed using an instrument grounded in education theory. Graduate entry students bring different life experiences to their learning to that of direct entry students. They experience ‘the shock of transition’ to clinical placement differently [17]. Their perceptions of the clinical learning environment may therefore be quite different to those of direct entry students. It is also advisable to test instruments when they are being used in new educational contexts [18].

In an attempt to better understand the student perspective of the instruments used to assess the clinical learning environment, students were asked their opinion on the questionnaires used. It is known that students do not always interpret questions in the manner that the questionnaire designer intended [19] and that this difference in interpretation has implications for the face validity of questionnaires [20]. Questionnaires therefore may not truly reflect students’ perspectives on their learning environments, particularly in a very complex environment such as a clinical workplace. It has also been found that students’ motivation to take part in these evaluation processes relates directly to their satisfaction with the process [21]. If students are not invested in the process they may engage in a superficial manner, which again can affect the validity of the measurement [22]. To our knowledge, exploring the student perspective of assessment instruments for the clinical environment has not yet been studied.

The overall aims of this study were to compare the DREEM and the more recently developed MCPI for the assessment of the clinical learning environment in a graduate entry medical student cohort and to explore student perceptions as to how the clinical educational environment is assessed. Specifically we wished:

1. To look at the correlation between MCPI and DREEM scores in a graduate entry population.
2. To compare the MCPI with the DREEM in a graduate entry population from the perspective of the student.
3. To investigate student perceptions as to how the clinical placement education environment is assessed.

## Methods

### Research context

The Graduate Entry Medical School (GEMS) at the University of Limerick offers a four year graduate entry medicine programme in Ireland. The average age of the students is 24, with a range of 21 to 43 years old. The students are 56% female. Approximately 70% have a primary Science degree, with 30% having varied undergraduate degrees. All students completed an honours degree prior to entry, and a smaller number of students have higher qualifications. Students are predominantly from EU countries at 68%, with 32% from non-EU countries, the majority of these students being from North America. Problem based learning forms the cornerstone of the first two years of the programme. In years 3 & 4 students are dispersed across a varied hospital network (one University Hospital, and several regional hospitals) and over 100 General Practices (Family Medicine Teaching Centres). This wide geographic spread coupled with the extended length of time spent by students in General Practice (18 weeks) can make for a more focussed, but on the other hand a more potentially isolated, learning experience. Exploring the quality of the clinical learning environment is therefore a priority.

### Participants

All third and fourth year graduate entry medical students on clinical placement in Academic Year 2015/2016 (*N* = 268) were invited to participate.

### Ethical approval and consent to participate

Ethical approval was granted by the Faculty of Education and Health Sciences Research Ethics Committee in the University of Limerick. The ethical review committee found that as an anonymous questionnaire was used, signed consent was not required as completing the questionnaire implied consent of the participant.

### Survey instrument

An online, anonymous survey comprising of both the DREEM and the MCPI instruments was created.

The DREEM is comprised of 50 items answered on a 5 point Likert scale [9] and its psychometric properties have previously been explored in this population [13]. Each item is scored from 0 to 4 with a maximum total score for the DREEM of 200. The authors of the DREEM provide a guide to interpreting its scores [23]. The DREEM has a maximum score of 200, 0–50 indicating a very poor learning environment, 51–100 indicating plenty of problems, 101–150 indicating more positive than negative, and 151–200 indicative of an excellent learning environment, as perceived by students. The DREEM has 5 subscales: *perceptions of learning (12 items), perceptions of teachers (11 items), academic self-perceptions (8 items), perceptions of atmosphere (12 items)* and *social self-perceptions (7 items)*. The DREEM is the current tool in use to assess the educational environment in the GEMS and is sent to students twice yearly with an additional open-ended question for comments. As this is the format the students are accustomed to, the open-ended question was retained for the purpose of this study.

The MCPI is comprised of 8 questions answered on a 7 point Likert scale with each question having an associated two-part open ended question [15]. Each item is scored from 0 to 6. The maximum score achievable on the MCPI is 48. The MCPI has 2 subscales: *learning environment (5 items)* and *training (3 items*).

Additional closed and open-ended questions were included in the survey to explore students’ perceptions of the DREEM and MCPI for giving feedback on their learning environment. Students were asked *‘To what extent has this questionnaire enabled you to give feedback on the important aspects of your clinical placement? Please rate your agreement with this statement: I have found completing this questionnaire worthwhile’* on a 7 point Likert scale (from 0 to 6) for each instrument. The MCPI was asked first, followed by the DREEM. The questions on suitability were delivered directly after completing each instrument. Participants were also asked to give free text comments on how useful they found the MCPI and the DREEM instruments with respect to delivering feedback on their clinical placement learning environment. Finally they were asked to outline their preference for how feedback on clinical placements should be sought; *‘What is your preference for giving feedback about your clinical placements? (For example, DREEM, MCPI, other online survey, one-to-one with tutor* etc.*)’* None of the free text boxes were compulsory.

Participants were not asked to provide demographic information as it was felt that this may risk deductive disclosure of student identities in placements with small numbers of students.

### Statistical analysis

Numeric variables (total DREEM score, total MCPI score, ratings) were tested for normality and summarised using mean (standard deviation) for normal distributions. Pearson’s correlation coefficient was used to measure the strength of the association between total DREEM score and total MCPI score for all students and for students on each placement with 10 or more students. GP practices were considered as one placement as it has been argued that, in terms of sociocultural aspects, the learning environments across practices are similar [24]. Pearson’s correlation coefficient was also used to measure the strength of the association between subscale scores for the DREEM and MCPI. The MCPI *learning environment* subscale *(5 items)* was compared with the DREEM *perceptions of atmosphere* subscale *(12 items)* as both look at the environment of the placements. The MCPI *training* subscale *(3 items*) was compared with the DREEM *perceptions of teachers* subscale *(11 items)* as both look at direct teaching to students. An absolute value of r of <0.2 was considered a very weak correlation; 0.2 to 0.39 weak; 0.40 to 0.59 moderate; 0.60 to 0.79 strong and 0.8 or above very strong. A paired t test was used to test for statistically significant differences in ratings given by students for how worthwhile they found both instruments for delivering feedback. A 5% level of significance was used for all tests. SPSS Version 21 for Windows was used for the analysis.

### Analysis of free text comments

Thematic analysis was used to analyse the free text comments provided via the open ended questions. Thematic analysis is ‘a method for identifying, analysing and reporting patterns within data’ and provides a rich and complex account of the data [25]. The approach as outlined by Vaismoradi [26] and the phases of data analysis as outlined by Braun and Clarke [25] were followed. This type of analysis divides data into smaller units, describing and interpreting them with a view to answering the research questions posed. The phases of analysis start with familiarising with data, generating initial codes, searching for themes, reviewing themes, defining and naming themes and finally producing a report. Nvivo software was used for analysing the free text qualitative data. This allowed for an audit trail to be created, allowing an external viewer to follow the logic of the analysis, thus ensuring transparency [27]. Reflexivity was achieved by using a reflective diary, tracking changes in perspective and allowing identification of personal biases.

## Results

### Response rates

The overall response rate to the questionnaire was 67% (*n* = 180), with a completed response rate for the MCPI instrument of 60% (*n* = 161) and for the DREEM instrument of 58% (*n* = 154). Only data from students who answered all questions was included in the analysis (*n* = 154). The year, location of placement of the respondents, and mean DREEM and MCPI scores are shown in Table 1. The mean DREEM score of 133 out of 200 (66.5%), and mean MCPI score of 32 out of 48 (66.7%) for all students indicated a more positive than negative learning environment.

**Table 1:** Response rates by year and placement, and total DREEM and MCPI scores

	Total number of students	Number of respondents (Response rate %)	Mean DREEM score (SD)^a^; % of best	Mean MCPI score (SD)^a^; % of best
Total	268	154 (58%)	133 (27.93); 66.5	32 (10.27); 66.7
Year 3	137	85 (62%)	132 (32.52); 66.0	32 (10.76); 66.7
Year 4	131	69 (52%)	133 (21.17); 66.5	32 (9.71); 66.7
GP Placement	97	59 (61%)		
University Hospital	68	42 (62%)		
Regional Hospital 6	26	9 (35%)		
Regional Hospital 5	20	7 (35%)		
Regional Hospital 2	19	13 (68%)		
Regional Hospital 1	18	8 (44%)		
Regional Hospital 4	14	9 (64%)		
Regional Hospital 3	7	7 (100%)		

^a^Not reported by site in order to preserve confidentiality

### Correlation between DREEM and MCPI scores

There was a strong, positive correlation between total DREEM and total MCPI scores (*r* = 0.71, *p* < 0.001).

There was a strong, positive correlation between the DREEM *perceptions of atmosphere* subscale and MCPI *learning environment* subscale (*r* = 0.63, *p* < 0.001). The correlation between subscales DREEM *perceptions of teachers* and MCPI *training* was moderate (*r* = 0.50, p < 0.001).

The correlations between the total DREEM and total MCPI scores across the placements and Year 3 and Year 4 was also examined (Table 2). Strong, positive correlations were found in all placements and in both years.

**Table 2:** Correlation between total DREEM and total MCPI by placement and year (placements with over 10 students)

CORRELATION	Pearson Correlation Coefficient r (*p*-value)
Total DREEM - Total MCPI	0.71 (p < 0.001)
DREEM *atmosphere -* MCPI *learning*	0.63 (p < 0.001)
DREEM *teachers -* MCPI *training*	0.50 (p < 0.001)
Placement Total DREEM - Total MCPI	
University Hospital (*n* = 42)	0.89 (p < 0.001)
Regional 2 (*n* = 13)	0.89 (p < 0.001)
GP Placement (*n* = 59)	0.63 (p < 0.001)
Year Total DREEM - Total MCPI	
Year 3 (*n* = 85)	0.77 (p < 0.001)
Year 4 (*n* = 69)	0.61 (p < 0.001)

### DREEM versus MCPI for enabling feedback delivery in a clinical learning environment – The student perspective

The mean rating for how worthwhile the students found completing the DREEM was 3.27 (SD 1.41) on a scale of 0 (worst) to 7 (best). The mean rating for the MCPI was 3.49 (SD 1.57) on the same scale, indicating a slightly higher positive rating than for the DREEM (mean difference 0.23, 95% confidence interval for the difference 0.01 to 0.45, *p* = 0.04).

The differences between the ratings by placement and year are given in Table 3.

**Table 3:** Mean difference between MCPI v DREEM for enabling feedback delivery by clinical placement and class year

	Mean DREEM (SD)	Mean MCPI (SD)	Mean difference (MCPI worthwhile minus DREEM worthwhile)
All students	3.27 (1.41)	3.49 (1.57)	.22 (0.04)
Placement			
GP Placement (n = 42)	3.3 (1.5)	3.3 (1.6)	.02 (0.01)
Regional 2 (n = 13)	3.7 (1.6)	4 (1.3)	.31 (0.17)
University Hospital (*n* = 58)	3.3 (1.2)	3.5 (1.4)	.21 (0.05)
Year			
Year 3 (*n* = 84)	3.4 (1.4)	3.6 (1.5)	.22 (0.05)
Year 4 (*n* = 68)	3.1 (1.3)	3.4 (1.6)	.25 (0.06)

#### Thematic analysis

Student comments on the DREEM and MCPI as methods for delivering feedback of the clinical educational environment are summarised in Table 4.

**Table 4:** Examples of the feedback on DREEM and MCPI for evaluating the clinical learning environment

DREEM	
Positive	“DREEM survey is handiest and most informative” “DREEM works well” “DREEM is better than MCPI”
Negative	“Too many questions in this section” “The questionnaire does not differentiate between the ward round tutors and the teaching tutors.” “Difficult to answer some of the questions relating to clinical tutors/teaching as the statements are broad and are not always correct for all tutors/teaching sessions.” “I don’t find all the questions asked on the DREEM survey relevant to the areas of the course that need improvement.” “I prefer the MCPI. I think the DREEM is good, but the statements are more vague and not always applicable to personal circumstances.”
MCPI	
Positive	“I think the questions are very well worded to tease our objective vs subjective problems on the side of the student. It encourages you to express your opinions.” “Ability to give feedback to specific segments, much much shorter than DREEM thus less off-putting for students…” “I liked the breakdown and structure and room for comments”
Negative	“This questionnaire addresses questions that you can’t necessarily talk about in DREEM. However I found the wording of the questions very difficult” “Perhaps a section on assessment of knowledge (3 separate segments of this questionnaire relate to clinical skills but none on acquisition of relevant information)” “It is useful in the sense that feedback is useful for the school, but not really a useful reflective process for me”
General	“They are very different surveys. They complement each other. One is not superior to the other, as they ask different questions.”

Free text comments reflected significant variability between students with respect to the preferred mechanism of feedback delivery. There was no single method of feedback cited by a majority of students as being the most useful or appropriate. The most frequently occurring preference was for one-to-one feedback, however a number of other students reported difficulty with providing feedback on a placement in a one-to-one setting, citing fear of repercussion. Taken all together, anonymous online surveys (i.e. DREEM, MCPI or other survey) was the most popular option chosen by students. A sample of different preferences expressed by students are shown below (Table 5).

**Table 5:** Student opinion on how best to provide placement feedback

“Online surveys are good. Face to face feedback falls short. I feel it never gets passed on”
“I think the school should talk to students, ideally in focus groups. These questionnaires are too restrictive, and are often given at a time that is inconvenient. Students’ thoughts and feelings about placements change over time. There should be opportunities to give feedback at various points throughout the semester. Students also need to be able to give feedback on a forum that they can be sure is anonymous or confidential.”
“One-to-one (or small group sessions) with an impartial/external person would be best.”
“I think a blend is good, but it’s good to be able to meet with a person and explain an issue properly.”
“Perhaps encourage the discussion board … to be used where students can post queries, staff can write replies to queries or comments to be seen by all students rather than anonymous surveys and emails back and forth….”
“….I think it would be good if consultants in hospitals asked us for feedback on our experience during our clinical placement with their teams.”
“There is a fear of being judged or deemed incompetent that may prevent students from talking to their clinical supervisor”

In order to look further at how the DREEM and MCPI performed the free text comments were analysed. There were 387 comments for the MCPI and 55 comments for the DREEM. When this data was analysed it was found that comments from the DREEM focussed on different areas to those from the MCPI, with much greater emphasis on interactions with academic staff and organisational aspects of the course, rather than the educational environment. In comparison comments from the MCPI tended to focus on the student experience while on placement, and allowed students to describe how they learned and found their way in a busy workplace. Themes emerging from analysis of the MCPI data were ‘learning at work’, ‘students in placement’, and ‘finding balance’. Under the theme *‘learning at work’* there were rich examples of how some students were well integrated into the workplace, with warm support and supervision, while others felt that they were left to flounder. Under the theme ‘*students in placement’* there were interesting comments on the nature of the roles of the student and the supervisors. Under the theme of ‘*finding balance’* was discussion from students about managing their own time and life outside medical school and also dealing with the inherent variability of clinical settings.

## Discussion

This study is the first study to compare the MCPI and the DREEM as instruments for the assessment of the clinical learning environment in a graduate entry cohort of medical students. Graduate entry students experience the ‘shock of transition’ to clinical practice differently to direct entry students [17]. These students have previous experience of higher level education and may have different insights into how feedback is sought. This is also the first study to investigate student perceptions as to how the clinical education environment is assessed.

### Assessing the clinical learning environment

A strong, positive correlation between total MCPI and DREEM scores was recorded indicating that scores in both instruments were related. Students tended to score both instruments high (or low) together when evaluating the clinical learning environment. The strength of this correlation however differs from that which was found in the only previous comparison study of the MCPI and DREEM [16]. In particular the correlations between the subscales are weaker. This finding may reflect the difference in the study populations with this present study, it is possible that graduate entry students, drawing on previous experiences, may interpret some questions in a different way to direct entry students which may explain the difference in the correlations found. Another potential explanation for weaker correlations in the graduate entry cohort is the difference in emphasis between the instruments. For example in the ‘*perceptions of teachers*’ subscale the DREEM asks about behaviours and attitudes demonstrated by teachers (‘*the course organisers are authoritarian’*), while the MCPI ‘*training*’ subscale focuses on the students experience while learning (*‘I was observed performing clinical skills on real patients’)*.

Unlike in previous work, this study investigated how acceptable both instruments were to students. This is of importance when considering which tool to use. For example, if an institution is considering using a shorter instrument that has better constructive feedback, it is important to know that it is equally acceptable to students. Free text comments indicated that the MCPI was the preferred method for assessing learning in the clinical setting for graduate entry medical students with this instrument providing a rich description of the clinical learning environment.

### Student perceptions on delivering feedback on the clinical learning environment

A strength of this study was investigating students perceptions of the measurement instruments employed. It is known that students are more motivated to participate in feedback exercises if they feel that they can give meaningful feedback [28]. Research has also demonstrated that response is more likely if the topic of the survey or questionnaire is of relevance to a respondent [29–32]. While students were neutral to slightly positive about the usefulness of both the MCPI and the DREEM as methods for enabling the delivery of feedback, the mean score for usefulness was slightly higher for the MCPI than for the DREEM. When placements were compared this difference was more apparent for regional placements. Reasons for this are not entirely clear, but may relate to the MCPI being more focussed on workplace issues and integration than the DREEM, and is therefore perceived by students to be more useful in smaller communities of practice rather than in large teaching hospital environments.

Looking at the comments from those who found the MCPI most worthwhile compared to the DREEM, students commented on “*how it allowed me to highlight the strengths and weaknesses of this rotation in comparison to other rotations*” and others “*It made me think more about my placement and what I should be getting from it*”. These comments suggest that those who found the MCPI most useful did in fact interpret the questionnaire in the manner intended. Those who found the MCPI least worthwhile relative to the DREEM made comments such as “*Only allows feedback on placement I am currently on and not other issues with course*”. Comments such as this may indicate that some students wish to comment on issues not pertaining to their placements, which is not the purpose of the MCPI. Overall, students appear to favour the MCPI which was described as “well worded” and “much shorter” over the DREEM which they describe as “vague” and having “too many questions”.

Further analysis of the free text comments also found that comments from the DREEM focussed on different areas to those from the MCPI. While the information in comments from the DREEM was useful, it did not reflect the learning environment as such. Rather it focussed on organisation and tutor aspects which may be better sought in a student evaluation of teaching (SETS) exercise. Comments from the MCPI were, on the other hand, more reflective of the type of learning environment students were working in, providing useful information on the clinical working environment and how students were integrated into it.

It is well known that students don’t always interpret questions in the manner intended [19] and that students rank issues differently to faculty in terms of importance [33]. One advantage therefore of seeking the, often overlooked [34], perspective of the students via comments is that it gives an insight into how students have difficulty interpreting some of the questions. For example, in this study students reported not being sure who the term ‘teachers’ referred to in the DREEM, that questions were ‘*too vague’* and finding it “*difficult to answer some of the questions relating to clinical tutors/teaching as the statements are broad and not always correct for all tutors/teaching sessions*”. It is clear therefore that understanding the perspective of the students, in particular their understanding with respect to what is being asked, is critical to interpreting correctly the findings of questionnaire data [19].

In their free text comments the students also identified a number of preferred mechanisms for giving feedback which included focus groups and semi-structured interviews suggesting a desire amongst students to provide more detailed qualitative feedback that that which a questionnaire allows. More detailed qualitative methods may therefore be helpful in gaining a deeper insight into student perceptions including the less tangible emotional aspects of their learning environment [35].

### Limitations

While one important limitation to this study is the response rate of 58% this, however, does exceed the reported average response rate of 29% for online surveys [36]. It is not known whether there are important differences between those who respond to online surveys and those who do not [37]. It is also likely that there is less risk of sampling bias with comparative research on two instruments, than when carrying out research on a single instrument.

Previous studies on the MCPI [15, 16] have also allowed examination of rating variation by each student across multiple placements. This current study was limited by the fact that it has only one placement rated for each student; therefore it wasn’t possible to assess variation between students across placements.

Another potential limitation is that there was no variability in the order in which the questionnaire was delivered. It is possible that this had an effect on response rate, questionnaire completion and on the comments given, although it did not appear to affect the completion rate of the questionnaires.

## Conclusion

The present study confirms that DREEM and MCPI total scores are strongly correlated in a graduate entry medical student cohort, the subscale scores are also moderately correlated. The strength of the correlation was weaker, particularly for the subscale scores, than that found in a previous study of direct entry medical students [16], possibly indicating that the strength of the association does not extrapolate to different student cohorts. There were methodological differences in the two studies that may also account for this.

Both instruments enabled the delivery of feedback on the clinical learning environment, the DREEM using 50 items, and the MCPI using 8. In contrast to previous work this study demonstrates the capacity of the MCPI to generate useful free text information which provides a rich description of the clinical learning environment. While students tended to favour the MCPI over the DREEM the free text comments made by the graduate entry students in this study may in fact indicate that the face validity of the MCPI is superior to that of the DREEM for this particular population cohort. The authors therefore recommend that the MCPI be used when assessing the graduate entry student learning experience in the clinical setting. Further exploration of MCPI qualitative free text comments in direct entry students would prove helpful in determining if this recommendation if transferable to all clinical medical student cohorts.

As the MCPI has the advantage of gaining richer detail on student experience, it represents a viable middle ground between detailed qualitative methods and the DREEM and is favoured over the DREEM by graduate entry medical students for the evaluation of learning in the clinical environment.

## Abbreviations

DREEM: Dundee ready education environment measure
MCPI: Manchester clinical placement index
